# Determining Pharmacological Selectivity of the Kappa Opioid Receptor Antagonist LY2456302 Using Pupillometry as a Translational Biomarker in Rat and Human

**DOI:** 10.1093/ijnp/pyu036

**Published:** 2015-01-29

**Authors:** Linda M. Rorick-Kehn, Jennifer W. Witcher, Stephen L. Lowe, Celedon R. Gonzales, Mary Ann Weller, Robert L. Bell, John C. Hart, Anne B. Need, Jamie H. McKinzie, Michael A. Statnick, Jeffrey G. Suico, David L. McKinzie, Sitra Tauscher-Wisniewski, Charles H. Mitch, Randall R. Stoltz, Conrad J. Wong

**Affiliations:** Eli Lilly and Company, Lilly Corporate Center, Indianapolis, Indiana (Drs Rorick-Kehn, Witcher, Lowe, Gonzales, Bell, Hard, Need, J. McKinzie, Statnick, Suico, D. McKinzie, Tauscher-Wisniewski, Mitch, and Wong); inVentiv Health Clinical, Ann Arbor, Michigan (Dr Weller); Covance Clinical Research Unit, Inc., Evansville, Indiana (Dr Stoltz).

**Keywords:** translational biomarker, pupillometry, kappa opioid receptor, dynorphin, LY2456302

## Abstract

**Background:**

Selective kappa opioid receptor antagonism is a promising experimental strategy for the treatment of depression. The kappa opioid receptor antagonist, LY2456302, exhibits ~30-fold higher affinity for kappa opioid receptors over mu opioid receptors, which is the next closest identified pharmacology.

**Methods:**

Here, we determined kappa opioid receptor pharmacological selectivity of LY2456302 by assessing mu opioid receptor antagonism using translational pupillometry in rats and humans.

**Results:**

In rats, morphine-induced mydriasis was completely blocked by the nonselective opioid receptor antagonist naloxone (3mg/kg, which produced 90% mu opioid receptor occupancy), while 100 and 300mg/kg LY2456302 (which produced 56% and 87% mu opioid receptor occupancy, respectively) only partially blocked morphine-induced mydriasis. In humans, fentanyl-induced miosis was completely blocked by 50mg naltrexone, and LY2456302 dose-dependently blocked miosis at 25 and 60mg (minimal-to-no blockade at 4–10mg).

**Conclusions:**

We demonstrate, for the first time, the use of translational pupillometry in the context of receptor occupancy to identify a clinical dose of LY2456302 achieving maximal kappa opioid receptor occupancy without evidence of significant mu receptor antagonism.

## Introduction

Although antidepressants are among the most widely prescribed medications, little progress has been made in improving clinical outcomes since the introduction of selective serotonin reuptake inhibitors almost 3 decades ago. Despite being effective therapies for some patients, monoamine-based medications (including selective serotonin reuptake inhibitors, older tricyclic antidepressants, and monoamine-oxidase inhibitors) suffer from delayed onset of antidepressant response and low remission rates (Berton and Nestler, 2006). Because of recent advances in molecular, behavioral, and genetic techniques, efforts are under way to develop novel nonmonoamine-based antidepressants that have the potential to dramatically improve antidepressant treatment response (Berton and Nestler, 2006; Insel, 2012).

Accumulating evidence suggests that selective kappa opioid receptor (KOR) antagonists may be beneficial in the treatment of mood and addictive disorders (Carr et al., 2010; Knoll and Carlezon, 2010; Harrison, 2013; Rorick-Kehn et al., 2014). A recent Phase 2 study demonstrated that ALKS-5461 (buprenorphine + ALKS-33), a formulation resulting in putative kappa antagonism, produced efficacy in treatment-resistant depressed patients with rapid onset of action (Harrison, 2013). In contrast, nonselective opioid antagonists, such as naltrexone, have shown no benefit on mood; rather, they produce dysphoria in certain preclinical models and patient populations (Mendelson et al., 1978; West and Wise, 1988). Preclinical data suggest that potential antidepressant-like effects of opioid antagonists are critically dependent upon KOR selectivity based on neurochemical and electrophysiological evidence that mu- and kappa-opioid systems oppose each other in the brain (Pfeiffer et al., 1986; Spanagel et al., 1992; Margolis et al., 2003). Therefore, it is hypothesized that the predominantly mu antagonist component of nonselective opioid antagonists may block the antidepressant effects of KOR antagonism. To test the hypothesis that kappa-selective opioid antagonists may have antidepressant effects, it is essential to identify in human subjects clinical exposures that selectively block KORs without evidence of mu opioid receptor (MOR) antagonism.

Pupillometry is a noninvasive research technique useful for studying the centrally mediated effects of opioids and other drugs in preclinical and clinical paradigms. As a translational biomarker, it is particularly useful for evaluating the potency, efficacy, and duration of action of opioid receptor agonists and antagonists (Knaggs et al., 2004; Lotsch, 2005; Matsoukova et al., 2011). Although MOR agonists produce miosis in humans, rabbits, and dogs, they produce mydriasis in other species, including rodents and cats (Klemfuss et al., 1979). Regardless of whether agonists produce pupillary constriction or dilation in the target species, it is well established that mu-preferring opioid antagonists such as naltrexone and naloxone reliably block the MOR agonist-induced effects in both animals and humans (Klemfuss et al., 1979; Greenwald et al., 1996; Jones et al., 2000).

**Figure 1.**
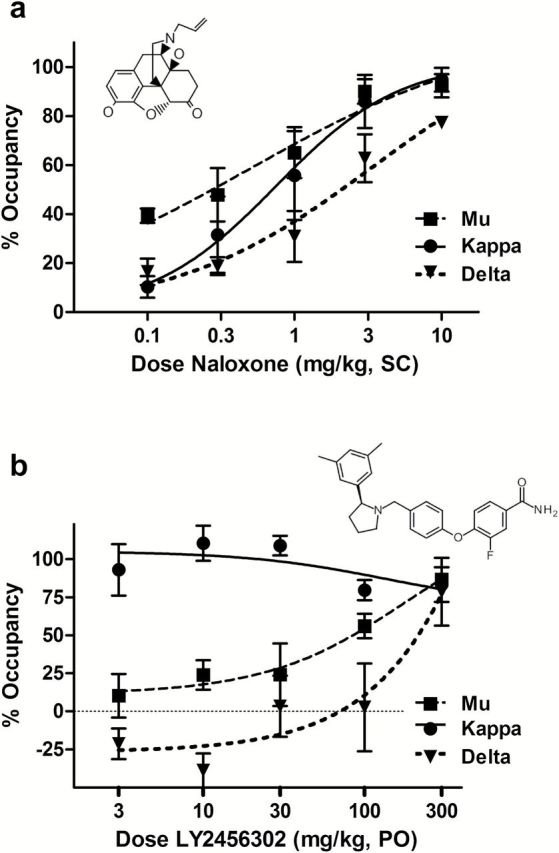
Dose-dependent in vivo receptor occupancy at putative mu, kappa, and delta opioid receptors (MOR, KOR, and DOR, respectively) by naloxone and LY2456302 60 minutes after administration. a, Naloxone showed dose- and concentration-dependent occupancy of putative opioid receptors in a manner consistent with its in vitro binding affinity, with MOR and KOR fully saturated at higher doses (3–10mg/kg). The doses at which 50% of receptors were occupied (ED_50_) at MOR, KOR, and DOR were 0.49, 0.75, and 3.45mg/kg, respectively. b, LY2456302 saturated putative KOR at all doses tested (3–300mg/kg). At higher doses, putative MOR and DOR occupancies were observed (ED_50_ values = 84.4 and 214.6mg/kg, respectively). LY2456302 selectively occupies KOR in the rat at doses <100mg/kg PO. Some engagement of MOR and DOR is evident at 100 and 300mg/kg (corresponding to brain exposures >473ng/g; see supplemental Table S1). Chemical structures of naloxone and LY2456302 are shown in insets.

LY2456302 ((S)-3-fluoro-4-(4-((2-(3,5-dimethylphenyl)pyrrolidin-1-yl)methyl)phenoxy)benzamide; chemical structure shown in Figure 1b inset) is a structurally novel, selective KOR antagonist with good central penetration and in vivo receptor occupancy that demonstrates efficacy in a number of preclinical models (Rorick-Kehn et al., 2014). In single- and multiple-dose clinical studies, LY2456302 was well tolerated up to doses of 35mg (Lowe et al., 2014). In a single-dose positron emission tomography study, LY2456302 demonstrated excellent central penetration and potent receptor occupancy in healthy human subjects, with full KOR occupancy at the 10-mg dose (Tauscher et al., 2012). Because MOR antagonist activity may be observed at higher doses of LY2456302, the current study was designed to identify the dose at which LY2456302 produces MOR antagonism in healthy subjects, thus confirming that lower doses remain selective for KORs.

Changes in pupil diameter in response to a mu-agonist challenge were measured as a pharmacodynamic (PD) biomarker in both rats and humans. Morphine-induced mydriasis was used in rats and fentanyl-induced miosis was used in humans to assess mu activity. Two fentanyl pupillometry studies were conducted in healthy volunteers, using a low dose of fentanyl (2 μg/kg, intravenously [IV]) that was previously well tolerated and produced robust miosis (Greenwald et al., 1996). In Study A, naltrexone was used to establish the mu-associated effects on miosis. In Study B, LY2456302 was administered to determine its effects on fentanyl-induced miosis.

## Preclinical Methods

### Animals

Male Sprague-Dawley rats (225–300g; Harlan, Indianapolis, IN) were pair-housed with ad libitum food/water and maintained on a 12-hour–light/–dark cycle. Experiments were conducted in accordance with Guidelines for Care and Use of Laboratory Animals under protocols approved by local Institutional Animal Care and Use Committee.

### Study Drugs

LY2456302 (Diaz-Buezo et al., 2009; Mitch et al., 2011) and GR103545 were synthesized at Lilly Research Laboratories (Indianapolis, IN). Naloxone HCl, naltrexone HCl, naltriben methanesulfonate hydrate (naltriben), and morphine sulphate (morphine) were purchased from Sigma Aldrich (St. Louis, MO). LY2456302 was dissolved in sterile water with the drop-wise addition of lactic acid and was orally dosed (3mL/kg). Naloxone and morphine were dissolved in 0.9% saline and dosed subcutaneously or intraperitoneally, respectively (1mL/kg). For in vivo receptor occupancy studies, nonlabeled naltrexone (10 µg/kg), naltriben (10 µg/kg), and GR103545 (1.5 µg/kg) were dissolved in saline and dosed IV, as tracers for mu, delta, and kappa receptors, respectively, in a single solution (0.5mL/kg).

### Receptor Occupancy

Occupancy of LY2456302 at opioid receptors was evaluated utilizing an in vivo rat model of central receptor occupancy. Microdoses of nonlabeled naltrexone (10 μg/kg), naltriben (10 µg/kg), and GR103545 (1.5 µg/kg) were used as tracers for MOR (Divin et al., 2009), delta opioid receptors (DORs) (Szekeres and Traynor, 1997), and KOR (Tomasi et al., 2013), respectively. Receptor occupancy was determined 60 minutes after an oral dose of LY2456302 (0, 3, 10, 30, 100, or 300mg/kg, n = 4/dose) or subcutaneous dose of naloxone (0, 0.1, 0.3, 1, 3, or 10mg/kg, n = 4/dose) by measuring striatal, thalamic, and cerebellar levels of each tracer by liquid chromatography coupled to tandem mass spectrometry (LC-MS/MS; Need et al., 2007). Total binding was represented by tracer levels in striatum (for DOR and KOR) and thalamus (for MOR). The cerebellum, which contains significantly lower densities of MOR, KOR, and DOR, was used for measuring nonspecific binding (Mansour et al., 1994).

Receptor occupancies were calculated using the ratio method described by Wadenberg and colleagues (2000) but substituting the tracer concentrations determined by LC-MS/MS for the radiolabeled tracer levels determined with scintillation spectrometry. The following equation was used:

$$\text{1}00*\left\{\text{1 }-\;\left[\left(\left({\text{ratio}}_{\text{t}}\;-\text{ 1}\right)⁄\left({\text{ratio}}_{\text{c}}\;-\text{ 1}\right)\right)\right]\right\}\;=\;\%\text{ occupancy}$$

Each “ratio” refers to the ratio of tracer in a target-rich brain area to the tracer level detected in the cerebellum. Ratio_t_ refers to animals treated with test drug and ratio_c_ refers to the average ratio in vehicle-treated animals.

### Tissue Preparation/Analysis for In Vivo Receptor Occupancy

Rat brain tissue samples were weighed and placed in conical centrifuge tubes on ice. Four volumes (wt/vol) of acetonitrile containing 0.1% formic acid was added to each tube. The samples were centrifuged after homogenization with an ultrasonic probe. Supernatant was diluted with sterile water in high pressure liquid chromatography (HPLC) injection vials for LC-MS/MS analysis. Tracer analysis was carried out using an Agilent 1200 HPLC (Agilent Technologies, Palo Alto, CA) and an API 3000 mass spectrometer (Applied Biosystems, Foster City, CA). The chromatographic separation employed Zorbax C18 column (Agilent Technologies, Wilmington, DE) gradient from 20% to 90% acetonitrile/water, each with 0.1% formic acid. The total HPLC run time was 3.5 minutes with an additional 2.0 minutes reequilibration time. Detection of small molecule tracers was accomplished by monitoring ion transitions 342.0:324.1, 416.0:398.2, and 414.0:343.0 mass:charge ratio for naltrexone, naltriben, and GR103545, respectively. Standards were prepared by adding known quantities of analyte to brain tissue samples from nontreated rats and processed as described above.

### Rat Pupillometry

Rats (n = 10/dose) were acclimated to the test room (30 lux, ambient lighting conditions) for 15 to 30 minutes. Pupil diameter was measured with a Nikon D80 digital camera affixed to a custom-built apparatus with a small vertical partition at a fixed distance (50cm) from the camera lens. The partition contained a circular opening (1cm) directly above a metal metric ruler also affixed to the partition. Pupil measurements were determined by briefly holding each rat (1–2 seconds) behind the partition so that the right eye was in the middle of the opening, during which time a digital photo was taken.

After a baseline pupil measurement, rats received 0, 3, 10, 30, 100, or 300mg/kg LY2456302. A postdose baseline pupil measurement was taken 60 minutes later, after which rats were immediately intraperitoneally dosed with 10mg/kg morphine. In a separate experiment, rats received 3mg/kg naloxone or vehicle 60 minutes before morphine administration. In each experiment, pupil measurements were taken at 10, 20, 30, 50, 70, and 90 minutes after morphine administration. A single measurement of pupil diameter (in millimeters) was taken at each time point and calculated as percent of baseline by dividing each measurement by the pretreatment baseline measurement (in millimeters). Pupil diameter was measured offline in a blinded manner.

### Statistics

Receptor occupancy data were calculated by Prism4 (GraphPad, San Diego, CA) and expressed as mean+standard error of the mean (SEM). Half maximal effective dose (ED_50_) values were calculated by fitting data to sigmoidal curves using nonlinear regression.

Maximum change in pupil diameter (MaxCPD) was calculated as the ratio of peak pupil diameter for each rat minus pretreatment pupil diameter over mean maximum change of the vehicle group and correlated with rat brain exposure from the receptor occupancy study (GraphPad; San Diego, CA).

For pupillometry, pupil diameter percent of baseline AUEC (area-under-the-effect-curve for pupil diameter in rats, and area-above-the-effect-curve for pupil diameter in humans; refers to the total calculated area of dilation in rats or constriction in humans) was computed for the 90-minute period using the trapezoidal rule. The AUEC values were log-transformed and analyzed using a 1-way analysis of variance model for bioequivalence with treatment of vehicle, LY2456302, and naloxone doses defined as a fixed-effect measure. Primary results were the least-squared mean contrasts (*t* test) for each LY2456302 dose and naloxone from vehicle. The least-squared mean difference and 90% confidence interval (CI) were back-transformed to obtain the mean ratio and corresponding 90% CI.

## Clinical Methods

Protocols and informed consent documents for Studies A and B were approved by the local Ethics Review Board. The studies were conducted in accordance with applicable laws and regulations of good clinical practice and ethical principles originating in the Declaration of Helsinki. Adverse events, clinical laboratory values, vital signs (blood pressure, pulse rate), and electrocardiogram results were monitored in both studies.

### Study Drugs

Naltrexone 50mg and placebo, supplied from Amide (a division of Mallinckrodt), were each given as 1 tablet in Study A. Fentanyl for Studies A and B was provided in vials as a citrate in water-soluble white crystalline powder from commercial drug product. When diluted, each milliliter of sterile aqueous solution contained a base of 50 μg fentanyl for IV use.

For Study B, LY2456302, provided by Eli Lilly and Company, was supplied as capsules containing 2 or 25mg LY2456302, with matching placebo capsules. After an overnight fast of ≥8 hours, LY2456302 or placebo capsules were given orally, with water, in the morning. Subjects fasted for at least 4 hours after receiving LY2456302 or placebo.

### Study Design

Study A was a randomized, subject- and investigator-blind, 3-period crossover study in healthy male subjects, ages 18 to 50 years, with a body mass index (BMI) ≥25 and ≤35kg/m^2^. Each period consisted of 3 days with ≥7 days washout between periods. An oral dose of naltrexone or placebo was administered on days 1 to 3. On day 3 (third period), approximately 1 hour after naltrexone or placebo administration, subjects were given an IV bolus of fentanyl 2.0 μg/kg or a total dose of 200 μg for subjects weighing ≥100kg.

Study B was a placebo-controlled, subject-blind, fixed-sequence, adaptive, crossover study with 5 treatment periods that included healthy males and females aged 18 to 65 years, with a BMI ≥18 and ≤32kg/m^2^. In Period 1, all subjects received a single dose of fentanyl and placebo; in Periods 2 through 5, subjects received a single dose of fentanyl and a single dose of LY2456302 at 4, 10, 25, or 60mg. Doses of LY2456302 were selected based on the safety and pharmacokinetic (PK) profile from a single ascending dose study in which doses from 2 to 60mg LY2456302 were administered to healthy volunteers (Lowe et al., 2014). Fentanyl (2.0 μg/kg or maximum total dose of 200 μg) was administered as a bolus IV injection approximately 2 hours after placebo or LY2456302, at the approximate maximal concentration of drug exposure (C_max_) of LY2456302, as previously determined (Lowe et al., 2014).

Blood sampling for determination of plasma concentrations of LY2456302 occurred at 0, 0.5, 1, 1.5, 2, 3, 4, 6, 8, 12, 24, 48, and 96 hours postdose.

### Bioanalytical Methods

Study B human plasma samples were analyzed at Advinus Therapeutics (Bangalore, India). Samples were analyzed for LY2456302 using LC-MS/MS. The lower limit of quantification was 0.20ng/mL and the upper limit of quantification was 202.70ng/mL. Interassay accuracy (percent relative error) ranged from −4.55% to 3.19%. Interassay precision (percent relative standard deviation) ranged from 2.10% to 4.76%.

### Pharmacokinetic Analyses

Study B plasma concentration-time data for LY2456302 were analyzed by noncompartmental methods using WinNonlin Enterprise 5.3. Actual sampling times were used in the estimation of LY2456302 PK parameters with predose times set to zero.

Log-linear trapezoidal rule method was used to estimate area under the plasma concentration vs time curve (AUC). Individual PK parameters estimated were C_max_, time of observed C_max_, AUC from time zero to last quantifiable time point (AUC_[0-tlast]_), AUC from time zero to infinity (AUC_[0-∞]_), half-life (t_1/2_), apparent clearance (CL/F), apparent volume of distribution at steady-state (V_ss_/F), and apparent volume of distribution (V_z_/F). Mean concentration values were calculated for graphical presentation.

### Pupillometry

Pupil diameter measurements were made with a validated infrared pupillograph (P2000SA Pupillometer, Procyon, Broomall, PA) using PupilFit software. Under scotopic light conditions (low luminosity – 0.04 lux), 3 to 5 pupil diameter measurements of each subject’s left and right eye were recorded and multiple scans (up to 5) were obtained at every 20-minute nominal time interval. Multiple measurements were aggregated to obtain a single value per nominal time point for each subject. Final mean pupil diameter and median acquisition time values were used in calculations.

For Study A, pupil diameter (millimeters) was measured on day 1 pre-naltrexone or -placebo, on day 3 pre-fentanyl, and then at 20-minute intervals for 120 minutes post-fentanyl administration. For Study B, pupil diameter (millimeters) was measured during each study period (for placebo and each dose of LY2456302) at pre-fentanyl administration and at 20-minute intervals for 180 minutes post-fentanyl administration.

Change in pupil diameter (CPD) from baseline over time was calculated to graphically display the time-course of pupil diameter. The apparent nadir of the pupil diameter time-course was observed and termed MaxCPD for each subject/treatment combination. The MaxCPD ratio was calculated as the ratio of treatment to placebo for the MaxCPD endpoint. The pupil diameter AUEC was computed using the trapezoidal rule for the pupil diameter (change from baseline) values.

### Pharmacokinetic/PD Relationship

The relationship between LY2456302 exposure (AUC_[0-∞]_) and effects on pupil diameter (MaxCPD and AUEC) was explored to characterize the exposure-response relationship. Modeling of the PK/PD relationships utilized maximum effect (E_max_) models in Nonmem (Version 7) and simple linear regression (rat data).

The E_max_ model utilized for the MaxCPD ratio analyses is shown in Equation 1. The E_0_ is the baseline ratio and is fixed to a value of 1, and E_max_ is the estimated maximal effect on MaxCPD ratio. The EC_50_ is the LY2456302 AUC that produces 50% of the E_max_.

$$Equation\;1:MaxCPD\;Ratio=E_{0}+\frac{\;E_{max}\times AUC}{\;EC_{50}+AUC}$$

The E_max_ model utilized for the AUEC analyses is shown in Equation 2. The E_0_ is the AUEC following fentanyl with a placebo dose for LY2456302, and the E_max_ is the estimated maximal effect on AUEC. The EC_50_ is the LY2456302 AUC that produces 50% of the E_max_. Intersubject variability was utilized for the E_0_ parameter. 

$$Equation\;2:\;\;AUEC=E_{0}+\frac{\;E_{max}\times AUC}{\;EC_{50}+AUC}\;$$

### Statistics

Study B data were independently analyzed. Pupil diameter AUEC values were log-transformed and analyzed using a repeated-measures mixed model with treatment of placebo and LY2456302 doses defined as a fixed effect and study period within each subject as a repeated measure. The primary contrast was each LY2456302 dose compared with placebo. The mean difference and CI were back-transformed to obtain the mean ratio and corresponding CI.

A meta-analysis was conducted on the log-transformed AUEC combining data from Study A and Study B. A linear mixed-effects model analyzed the natural log (AUEC) with the random effect of subject and fixed effects of clinical trial (study A and study B) and the treatment effects (placebo-Study A, placebo-Study B, LY2456302 doses and naltrexone 50-mg dose). The primary results were back-transformed to least-squares means with the corresponding 95% mean CI. Pair-wise mean contrasts and corresponding *P*-values were computed between all LY2456302 doses, naltrexone, and placebo-Study A compared with placebo-Study B.

### Sample Size Statistics

Assuming an equivalence range of +20%, a sample size of 10 subjects provided 80% probability that the 90% CI of the overall change in pupil diameter by LY2456302 dose vs placebo would not include 1 (ie, would detect reversal of mu-agonist–induced effects). Exploratory analysis of Study A revealed that the mean ratio of pupil diameter of naltrexone:placebo under scotopic light conditions during a 3-hour post-fentanyl dose was 1.38, with a within-subject coefficient of variation of 11%. Based on these findings, the sample size of 10 subjects was sufficient for detecting reversal of mu-agonist–induced effects by LY2456302, as identified when the 90% CI of the mean ratio does not include the value of 1.

## Results

### Rat In Vivo Receptor Occupancy

Naloxone showed a dose- and concentration-dependent increase in opioid receptor occupancy consistent with its in vitro binding affinity, with MOR and KOR fully saturated at higher doses (3–10mg/kg; Figure 1; supplementary Table S1). The doses at which 50% of MOR, KOR, and DOR were occupied (ED_50_) were 0.49, 0.75, and 3.45mg/kg, respectively (Figure 1a).

In rats, LY2456302 saturated KOR at all doses tested (3–300mg/kg) and demonstrated dose- and concentration-dependent increases in occupancy of MOR and DOR receptors (Figure 1b), consistent with its in vitro binding affinity (Ki at recombinant human receptors = 0.81, 24.0, and 155nM for KOR, MOR, and DOR, respectively; Rorick-Kehn et al., 2014). The increasing levels of MOR and DOR occupancy at higher doses (ED_50_ = 84.4 and 214.6mg/kg, respectively) were driven by proportional increases in plasma and brain exposure of LY2456302 (supplementary Table S1). Results demonstrate that LY2456302 selectively occupies KORs in rat brain with high potency and selectivity in the pharmacological dose range (1–10mg/kg; Rorick-Kehn et al., 2014), whereas MOR occupancy was observed at LY2456302 doses and exposures ≥10-fold higher than required for preclinical efficacy.

### Clinical Demographics

Subject demographics are presented in supplementary Table S2. Study A enrolled 15 male subjects (13 completed), and 14 received at least 1 dose of naltrexone; 1 subject received only placebo. Both noncompleters cited personal reasons for early discontinuation. Subjects had a mean age of 32.6 years (range 20–44 years) and mean BMI of 30.20kg/m^2^ (range 25.95–34.84kg/m^2^). Study B enrolled 11 (6 males) subjects with a mean age of 39.8 years (range 21–52 years), mean weight of 72.3kg (range 49.8–95.7), and mean BMI of 24.6 (range 19.9–27.3). Ten subjects received at least 1 dose of LY2456302; 1 subject received only placebo plus fentanyl. Seven subjects completed Study B; of the 4 subjects who did not, reasons for early discontinuation included subject decision (2), protocol violation (1), and lost to follow-up (1).

### Pharmacokinetics

In Study B, after single-dose administration, LY2456302 was rapidly absorbed with peak plasma concentrations occurring at 1.5 to 4 hours postdose (Table 1). Pharmacokinetic results were compared with data from a previous single-ascending dose study (Table 1). Following coadministration with fentanyl, there was higher apparent clearance (CL/F) and shorter half-life (t_1/2_) for the 4-, 10-, and 25-mg doses compared with LY2456302 alone. In contrast, at the 60-mg dose, CL/F was comparable between LY2456302 alone and with fentanyl. Overall, the PK of LY2456302 was comparable when administered alone or with fentanyl. Exposure estimates for each subject were used in the exposure/response pupillometry analyses for pupillometry.

**Table 1.** Noncompartmental Pharmacokinetic Parameters Following a Single Oral Dose of LY2456302 Alone and After Administration of Fentanyl in Healthy Subjects

	LY2456302^a^ Geometric Mean (CV%)					LY2456302 plus Fentanyl Geometric Mean (CV%)			
LY2456302 dose, mg	2	4	10	25	60	4	10	25	60
n	6	8	7	7	7	7	10	10	10
C_max_, ng/mL	4.20 (25)	11.6 (26)	27.6 (26)	72.2 (33)	129 (28)	6.71 (94)	23.4 (27)	46.3 (49)	137 (49)
t_max_^c^, h	1.50 (1.50–4.00)	2.01 (1.50–3.00)	1.50 (1.50–3.00)	2.00 (1.33–3.00)	2.42 (1.50–3.08)	3.00 (2.00–4.00)	2.02 (1.50–3.00)	2.57 (1.50–4.00)	3.00 (1.50–4.00)
t_1/2_, h	21.3 (62)	32.3 (42)	38.5 (22)	34.1 (32)	35.1 (27)	21.4 (55)	22.5 (39)	25.1 (30)	25.1 (36)
AUC(0-∞), ng⋅h/mL	45.6 (46)	139 (37)	348 (36)	924 (36)	1730 (48)	91.4 (43)	268 (31)	561 (31)	1700 (30)
CL/F, L/h	43.9 (46)	28.7 (37)	28.7 (36)	27.1 (36)	34.8 (48)	43.8 (43)	37.4 (31)	44.6 (31)	35.2 (30)
V_z_/F, L	1350 (16)	1340 (32)	1590 (34)	1330 (42)	1760 (35)	1350 (49)	1210 (30)	1610 (34)	1270 (27)
Vss/F, L	1070 (22)	1020 (27)	1160 (25)	977 (34)	1320 (33)	1130 (44)	904 (26)	1160 (39)	973 (27)

Abbreviations: AUC(0-∞), area under concentration vs time from zero to infinity; C_max_, maximum observed drug concentration; CV, coefficient of variation; t_max_, time to maximum observed drug concentration; t_1/2_, half-life associated with the terminal rate constant (λ_z_) in noncompartmental analysis; CL/F, apparent total body clearance of drug calculated after extra-vascular administration; V_z_/F, apparent volume of distribution during the terminal phase after extra-vascular administration; V_ss_/F, apparent volume of distribution at steady state after extra-vascular administration.

^a^ Data for LY2456302 alone is included from Lowe, Wong, Witcher, Gonzales, Dickinson, Bell, et al. (unpublished data) for comparison.

^b^ LY2456302 plus fentanyl data obtained in Study B. Fentanyl administered 2h after LY2456302.

^c^ Median (range).

### Safety

No serious adverse events were reported in Studies A or B. In Study A, the most frequent adverse event (AE) reported during and after fentanyl administration was itching (n=8). In 3 subjects, nausea led to emesis at approximately 200 μg of fentanyl.

In Study B, a total of 75 AEs were reported by 11 subjects who received ≥1 dose of LY2456302 and/or placebo (supplementary Table S3). The most common AEs considered related to LY2456302 (occurring >1 incidence) were headache (4 events) and nausea (2 events), all mild in severity. After the 25-mg LY2456302 dose, 1 subject experienced severe vomiting and flushing, both short-lived and likely related to fentanyl treatment. Most AEs deemed not related to study drug were consistent with known fentanyl effects. There were no clinically significant values, changes, or trends observed in clinical laboratory data, including neurohormones (cortisol, prolactin, and luteinizing hormone), vital sign values, or electrocardiogram parameters.

### Pupillometry

**Figure 2.**
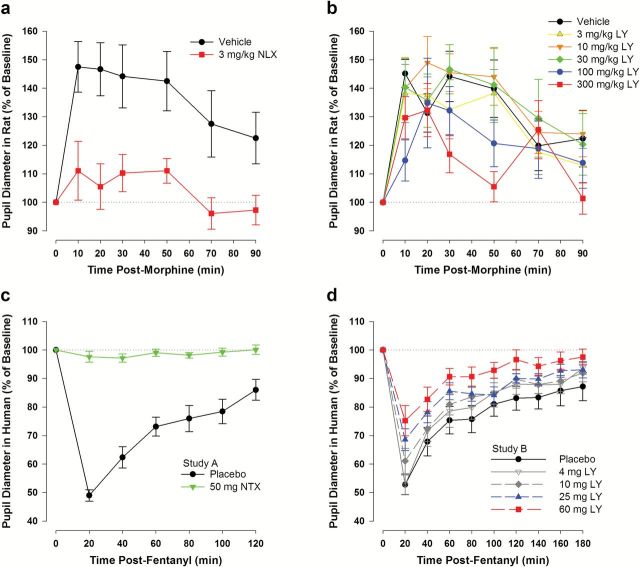
Effect of naloxone (NLX) and LY2456302 (LY) on morphine-induced mydriasis in the rat and naltrexone (NTX) and LY2456302 on fentanyl-induced miosis in healthy human subjects. a, Naloxone (3mg/kg subcutaneously) completely blocked morphine-induced mydriasis in the rat, indicating full blockade of mu opioid receptor (MOR). b, LY2456302 produced modest, but significant, blockade of morphine-induced mydriasis in the rat at the highest doses tested (100 and 300mg/kg orally), consistent with 56% and 87% MOR occupancy, respectively. At lower doses, LY2456302 showed no evidence of MOR blockade. c, Naltrexone (50mg) completely blocked fentanyl-induced miosis in healthy human subjects. d, LY2456302 produced dose- and concentration-dependent blockade of fentanyl-induced miosis in healthy subjects, with statistically significant effects at 25 and 60mg. Doses of 4 and 10mg LY2456302 did not significantly affect fentanyl-induced miosis. Data in each panel represent the mean (±SEM) pupil diameter, expressed as a percent of baseline pupil diameter. See supplementary Table S4 for raw pupil diameter values (in millimeters).

**Figure 3.**
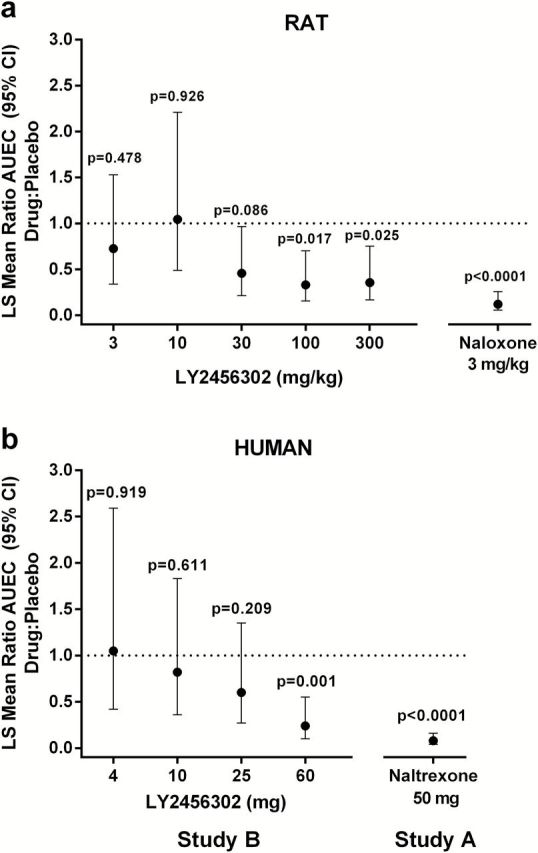
Statistical analysis of the least squares geometric means of the area under (or above) the effect curve (AUEC) of pupil diameter in rats and healthy subjects. Mean estimates of AUEC ratio of drug: placebo across dose levels showed a dose-response relationship in rats (a) and healthy subjects (b). Dotted line indicates a ratio of 1, or no effect on fentanyl-induced mydriasis or miosis, respectively.

Pupil diameter was used as a PD measure to assess the ability of LY2456302 to block morphine-induced mydriasis in rats and fentanyl-induced miosis in humans. As illustrated in Figure 2a, morphine-induced mydriasis in rats was completely blocked by 3mg/kg naloxone, a dose that fully occupied MOR (CI of mean difference relative to vehicle: 0.057, 0.256), *P*<.001; also confirmed by AUEC analysis) (Figure 3a). At doses ≤30mg/kg, LY2456302 did not block morphine-induced mydriasis (Figure 2b; all *P*>.05); however, at higher doses (100 and 300mg/kg), LY2456302 partially, but significantly, blocked morphine-induced mydriasis (Figure 2b with bio-equivalence to vehicle and confirmed by AUEC analysis; see Figure 3a of 90% mean difference CI relative to vehicle; 100mg/kg: [CI: 0.156, 0.703), *P*=.017; 300mg/kg: [CI: 0.168, 0.753], *P*=.025). Hence, MOR occupancy in the range of 60% to 85% produced partial PD effects, whereas >90% MOR occupancy was required to fully block morphine-induced mydriasis in rats under the present conditions. Raw pupil diameter means (±SEM) are reported in supplementary Table S4.

In Study A, naltrexone administration on day 1 (without fentanyl) did not influence pupil diameter under scotopic lighting conditions (overall treatment effect, *P*=.373; data not shown). Fentanyl administration on day 3 produced significant miosis in healthy subjects (after preadministration of placebo) with a maximum effect observed at 20 minutes post-fentanyl administration (the first measurement post-fentanyl; overall bioequivalence test: *P*<.001). Preadministration of naltrexone fully blocked the fentanyl-induced miosis (bioequivalence test: *P*<.001; Figure 2c, see also LS Mean Ratio AUEC analysis below and in Figure 3b). Raw pupil diameter means (±SEM) are reported in supplementary Table S4.

In Study B, fentanyl produced a rapid onset of miosis with an apparent nadir observed at 20 minutes for all groups, consistent with Study A (Figure 2d; raw pupil diameter means (±SEM) are reported in supplementary Table S4). The placebo group (fentanyl alone) had an approximately 3-mm decrease in pupil diameter at the nadir or an approximately 50% decrease from baseline. Miosis gradually dissipated during the 180-minute post-fentanyl dosing. LY2456302 produced dose- and concentration-dependent blockade of fentanyl-induced miosis in healthy subjects (bioequivalence test: *P*=.01; see also LS Mean Ratio AUEC analysis below and in Figure 3b). Doses of 4 and 10mg LY2456302 did not affect fentanyl-induced miosis, whereas a moderate degree of blockade was observed at the 25- and 60-mg doses (Figure 2d).

The area-under-the-pupil-diameter vs time curve, termed AUEC, was also used to evaluate the exposure- and dose-response relationships. Analysis of AUEC across dose levels and comparison with placebo showed a dose-response relationship. In Figure 3, a ratio of 1 indicates no effect on fentanyl-induced miosis (or mydriasis), and decreasing ratios indicate more blockade of mu-agonist–induced miosis (or mydriasis). A similar relationship was observed for LY2456302 in rat and human. Naloxone (rat study) and naltrexone (human Study A) produced similar results, with both having very low ratios or nearly complete blockade of mu-agonist–induced effects. In rats and humans, the naloxone and naltrexone ratios were generally lower than ratios for all LY2456302 doses. Meta-analysis showed that all LY2456302 doses were significantly different from naltrexone (data not shown).

**Figure 4.**
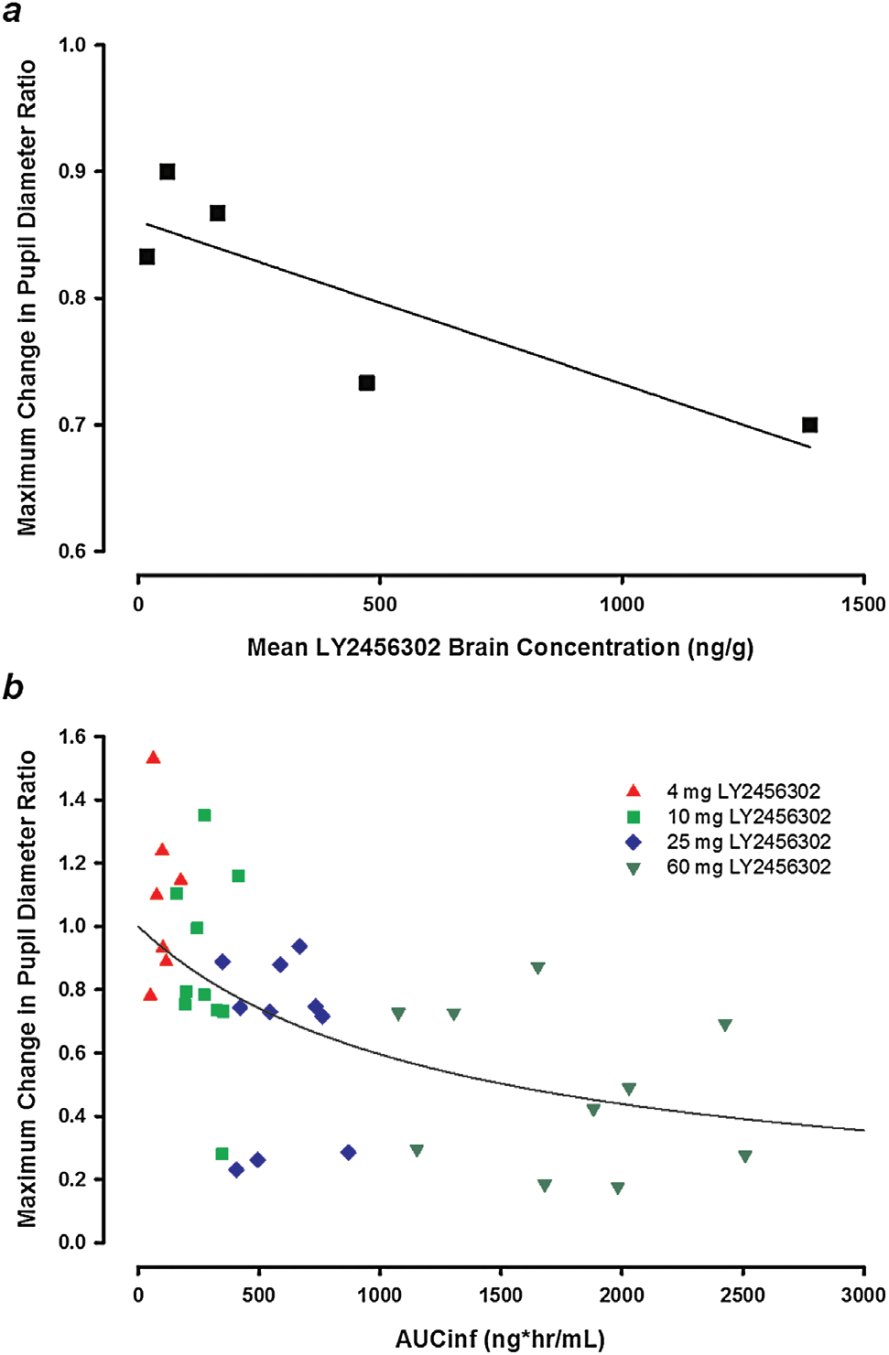
Maximum change in pupil diameter ratio. a, Maximum change in pupil diameter (MaxCPD) ratio in rats (drug:vehicle) is inversely correlated with LY2456302 brain exposure, indicating that, at higher exposures, LY2456302 reduces the magnitude of morphine-induced mydriasis observed. b, MaxCPD ratio in humans (treatment:placebo) is inversely related to LY2456302 exposure and described by the E_max_ (the estimated maximal effect on MaxCPD ratio) model (E_0_ [the baseline ratio]=1.00 [fixed]; E_max_=−0.918 [36.1%]; EC_50_ [the AUC that produces 50% of the E_max_]=1270 [72.8%]).

The nadir of the pupil diameter curve, termed the maximum change in pupil diameter from baseline (MaxCPD), was used as a pupillometry endpoint to further evaluate the PK/PD relationship. In rats, the MaxCPD was the peak of the pupil diameter curve due to the observed mydriasis in rats, whereas in humans it was the nadir due to observed miosis. The MaxCPD ratio was calculated using the nadir (or peak) change from baseline for each treatment compared with placebo. A ratio of 1 indicates no blockade of fentanyl-induced miosis/mydriasis, whereas a ratio approaching zero indicates complete blockade of miosis/mydriasis. An exposure-response relationship for MaxCPD was observed in both rats and humans (Figure 4). The exposure endpoint was brain concentration in rats and plasma AUC in humans. As predicted, the MaxCPD in rats was inversely correlated with LY2456302 brain concentrations (Figure 4a). The exposure-response relationship in healthy humans (Figure 4b) was best described using a E_max_ (the estimated maximal effect on MaxCPD ratio) model (E_0_ [the baseline ratio]=1.00 [fixed]; E_max_=–0.918 [36.1%]; EC_50_ [the AUC that produces 50% of the E_max_]=1270 ng*h/mL [72.8%]). The MaxCPD ratio would approach zero at the maximum effect, based on the model parameters. Based on the model fit, the mean AUC for the 60-mg dose (1700 ng*h/mL) corresponded to a MaxCPD ratio estimate of 0.47, indicating that approximately one-half of the fentanyl-induced miosis was blocked at this dose. The EC_50_ estimate was 1270 ng*h/mL, suggesting the 60-mg dose produced some moderate blockade of fentanyl-induced miosis, as measured by MaxCPD ratio. Therefore, the maximum effect for the MaxCPD ratio is expected to be achieved at an LY2456302 dose outside the dose range evaluated.

The AUEC exposure-response relationship was evaluated using an E_max_ model (supplementary Figure S1). This relationship was similar to the dose-response relationship described above for MaxCPD. Similar to MaxCPD (Figure 4), the E_max_ model parameters suggested that doses of 25 and 60mg produced some moderate effect, as measured by AUEC, and the maximum effect would be expected at an LY2456302 dose outside the dose range evaluated.

## Discussion

We demonstrate here for the first time that the KOR antagonist, LY2456302, produced dose- and concentration-dependent occupancy of MOR in rats at doses and concentrations ≥10-fold higher than those producing efficacy in preclinical models (Rorick-Kehn et al., 2014). Moreover, we used pupillometry as a translational biomarker in rats and humans to determine doses of LY2456302 necessary to reverse MOR agonist-induced effects, thereby confirming that lower doses remain KOR selective. The higher exposures of LY2456302 required to block MOR-agonist–induced effects on pupil diameter were consistent with its in vitro binding affinity and preclinical in vivo pharmacology (Rorick-Kehn et al., 2014). This finding is noteworthy, because there are currently no KOR-selective antagonists available for clinical evaluation, although the mixed opioid compound buprenorphine, in combination with a mu-preferring antagonist to block its mu partial agonist properties (ALKS-5461), produced significant improvement in depressed patients after 1 week of dosing (Harrison, 2013). Importantly, our findings demonstrate that LY2456302 selectively blocks KOR without evidence of significant MOR antagonism within the dose range of 4 to 10mg in humans (1–30mg/kg in rats).

In the rat receptor occupancy study, naloxone produced dose- and concentration-dependent MOR occupancy, with an ED_50_ of 0.49mg/kg, consistent with reported ED_50_ values for blockade of morphine-induced analgesia in rodents (Vaccarino et al., 1988) as well as efficacy in treating opioid overdose and displacement of ^11^C-diprenorphine binding in humans (Melichar et al., 2003). In the rat pupillometry study, a 3-mg/kg dose of naloxone that produced 90% MOR occupancy completely blocked morphine-induced mydriasis. The highest doses of LY2456302 tested (100 and 300mg/kg) produced 56% and 87% MOR occupancy, respectively, and partially blocked morphine-induced mydriasis. The reason for the less robust blockade of morphine-induced mydriasis by LY2456302 is unknown but may be related to the greater overall variability observed in that study.

Our data are consistent with a parametric pupillometry study demonstrating that while morphine dose-dependently increased mydriasis in rats, fluctuations in pupil diameter also dramatically increased in a dose-related manner (Klemfuss et al., 1979). The apparent incomplete blockade may also be related to the relative contributions of MOR and KOR in modulating pupil dynamics. Whereas MOR agonists are well known to regulate pupil diameter, KORs are known to modulate intraocular pressure (Russell and Potter, 2002; Rasmussen et al., 2007). Specifically, KOR agonists reduce intraocular pressure, an effect that is blocked by KOR antagonists (Russell et al., 2000; Russell and Potter, 2002; Rasmussen et al., 2007). Therefore, it is conceivable that LY2456302 effects on intraocular pressure may alter the ability of morphine to produce pupillary changes, such that greater occupancy at MOR is required to overcome alterations in pupil dynamics produced by changes in intraocular pressure. Further studies will be required to determine whether super-saturation of KOR significantly alters mu-agonist–mediated regulation of pupil dynamics.

Consistent with preclinical studies, single oral doses of LY2456302 demonstrated rapid central penetration and potent receptor occupancy in healthy human subjects, with 75% to 100% KOR occupancy at doses of 2 to 25mg, as measured by displacement of the KOR-selective antagonist positron emission tomography tracer, ^11^C-LY2795050 (Tauscher et al., 2012). Demonstration of potent KOR occupancy after oral dosing in human subjects provides greater confidence that the clinical hypothesis that KOR-selective antagonists have antidepressant effects will ultimately be tested in Phase 2 studies. It is believed that antidepressant effects of opioid antagonists are critically dependent upon KOR selectivity over other opioid receptor subtypes due to the dysphoria produced by nonselective opioid antagonists, which may attenuate the antidepressant effects of KOR antagonism (Mendelson et al., 1978; West and Wise, 1988). Therefore, we used translational pupillometry techniques to identify clinical doses of LY2456302 that do not exhibit significant antagonism at MOR, which will be used in future efficacy studies.

In our studies in healthy subjects, fentanyl produced robust miosis under scotopic lighting conditions, consistent with previous reports that dim lighting facilitated optimal detection of pupillary changes by opiates (Weinhold and Bigelow, 1993). The fentanyl-induced miosis was completely blocked by 50mg naltrexone. LY2456302 doses of 25 and 60mg produced minimal to moderate, but statistically significant, blockade of fentanyl-induced miosis, indicating MOR antagonism. Based on observed MOR activity at 25- to 60-mg doses, a clinical dose of 4 to 10mg LY2456302 would provide robust and selective KOR occupancy. Importantly, this dose range was well tolerated in healthy subjects (Lowe et al., 2014). Therefore, the dose- and exposure-response relationships measured here can inform Phase 2 dose selection to avoid doses with notable MOR blockade.

The PK profile of LY2456302 was well characterized after single doses and compared with similar data from another study (Lowe et al., 2014). Some minor differences in mean PK profiles were noted between LY2456302 alone and when co-administered with fentanyl, most likely because of variability between subjects and periods and not related to drug-drug interactions. Overall, LY2456302 was well tolerated in healthy subjects.

In conclusion, LY2456302 maintains selectivity for KOR within the 4- to 10-mg dose range in healthy volunteers. In conjunction with receptor occupancy, pupillometry represents an excellent translational tool to determine the pharmacological selectivity of novel KOR antagonists, which can be used to test the clinical hypothesis that KOR-selective antagonists may produce antidepressant effects. Here, we provide a model for using a hypothesis-driven approach in developing translational biomarkers that have great impact on guiding dose selection in early clinical trials, based on a clear understanding of target engagement and pharmacological selectivity.

## Supplementary Material

For supplementary material accompanying this paper, visit http://www.ijnp.oxfordjournals.org/

## Statement of Interest/Disclosure

L.M.R.-K., J.W.W., S.L.L., C.R.G., R.L.B., J.C.H., A.B.N., J.H.M., M.A.S., J.G.S., D.L.M., S.T.-W., C.H.M., and C.J.W. are employees of, and stockholders in, Eli Lilly and Company at the time the experiments were conducted. M.A.W. is an employee of inVentiv Health Clinical. R.R.S. is an employee of Covance.

## Supplementary Material

http://www.ijnp.oxfordjournals.org/
