# Supplementary material for: Determining Pharmacological Selectivity of the Kappa Opioid Receptor Antagonist LY2456302 Using Pupillometry as a Translational Biomarker in Rat and Human
Source: Int J Neuropsychopharmacol. 2015 Jan 29;18(2):pyu036. doi: 10.1093/ijnp/pyu036 (PMC4368892; doi:10.1093/ijnp/pyu036)
Supplement: http://www.ijnp.oxfordjournals.org/ [file ijnp_pyu036_index.html]

Supplementary Data | International Journal of Neuropsychopharmacology

## Supplementary Data

Data files

**Files in this Data Supplement:**

- Supplementary Data - Supplementary Data
- Supplementary Data - Supplementary Data
