# Supplementary material for: Determining Pharmacological Selectivity of the Kappa Opioid Receptor Antagonist LY2456302 Using Pupillometry as a Translational Biomarker in Rat and Human
Source: Int J Neuropsychopharmacol. 2015 Jan 29;18(2):pyu036. doi: 10.1093/ijnp/pyu036 (PMC4368892; doi:10.1093/ijnp/pyu036)
Supplement: http://www.ijnp.oxfordjournals.org/ [file SUPPLEMENTARY.docx]

**SUPPLEMENTARY FIGURE LEGEND:**

**Supplementary Fig S1. Area below baseline curve vs LY2456302 exposure - AUC(0-inf) in Study B.**

The figure shows the PK/PD relationship for the AUEC endpoint. An exposure-response relationship was observed for this endpoint, and it was similar to the dose-response relationship described in the full text. An E_max_ model suggests that the E_max_ is not yet reached over the range of this data. The AUEC value at the E_max_ is estimated to be -67 mm*min, the mean AUC for the 60-mg dose (1700 ng*hr/mL) corresponds to an AUEC estimate of 108 mm*min. Therefore, the maximum effect would be achieved at an LY2456302 dose outside the dose range evaluated. The EC_50_ estimate suggested the doses of 25 mg and 60 mg have some moderate effect as measured by AUEC.

**Supplementary Table S1**. Drug levels in rat striatum and plasma 60 minutes after a PO dose of LY2456302 (0, 3, 10, 30, 100, or 300 mg/kg) or SC dose of naloxone (0, 0.1, 0.3, 1, 3, or 10 mg/kg) measured by liquid chromatography coupled to tandem mass spectral detection (LC-MS/MS)

|  | | **LY2456302** | | | | | **Naloxone** | | | | |
| --- | --- | --- | --- | --- | --- | --- | --- | --- | --- | --- | --- |
|  |  | **Brain** | | | **Plasma** | | **Brain** | | | | |
| **Dose (mg/kg)** | **n** | **ng/g** | **SEM** | **ng/mL** | | **SEM** | | **Dose (mg/kg)** | **n** | **ng/g** | **SEM** |
| **3** | 3 | 18.3 | 2 | 11.3 | | 1 | | **0.1** | 4 | 18.5 | 1.09 |
| **10** | 3 | 60.7 | 3.7 | 34.0 | | 3 | | **0.3** | 4 | 44.1 | 3.14 |
| **30** | 3 | 164.3 | 56.6 | 76.7 | | 13 | | **1** | 4 | 118.2 | 9.54 |
| **100** | 3 | 472.7 | 131.1 | 235.3 | | 95 | | **3** | 4 | 244.7 | 49.95 |
| **300** | 3 | 1388.7 | 307.5 | 660.7 | | 154 | | **10** | 4 | 1659.4 | 104.62 |
| Abbreviation: SEM, standard error of the mean. | | | | | | | | | | | |

**Supplementary Table S2. Subject Demographics for human studies A and B**

| **Parameter** | **Study A** | **Study B** |
| --- | --- | --- |
|  | **N=15** | **N=11** |
| **Mean Age (years)** | 32.6 | 39.8 |
| **Range (years)** | 20–44 | 21–52 |
| **Sex (n)** |  |  |
| **Male**  **Female** | 15  0 | 6  5 |
| **Race (n)**  **White**  **Black** | 9  6 | 9  2 |
| **Ethnicity (n)**  **Hispanic or Latino**  **Not Hispanic or Latino** | na ^a^  na ^a^ | 1  10 |
|  |  |  |
| **Mean BMI (kg/m^2^)** | 30.2 | 24.6 |
| **Range (kg/m^2^)** | 26.0–34.9 | 19.9–27.3 |

^a^ not available

Abbreviations: BMI, body mass index.

**Supplementary Table S3.** **Study B Adverse Events**. Frequency of subjects with treatment-emergent adverse events all causality and related to study treatment

|  | **Number of Adverse Events (Number of Subjects with Adverse Event)** | | | | | | | | | | | | | | | |
| --- | --- | --- | --- | --- | --- | --- | --- | --- | --- | --- | --- | --- | --- | --- | --- | --- |
|  | **Placebo with Fentanyl** | | | | **LY2456302 with Fentanyl** | | | | | | | | | | | |
|  | **(n=11)** | | | | **4 mg  (n=7)** | | | | **10 mg  (n=10 )** | | **25 mg  (n=10 )** | | | **60 mg  (n=10 )** | |  |
|  | **Total** | | | **Related** | **Total** | **Related** | | | **Total** | **Related** | **Total** | **Related** | | **Total** | **Related** | **Total AEs** ^a^ |
| **Headache** | 2 (2) | | |  | 2 (2) | 1 (1) | | | 2 (2) |  | 6 (5) | 2 (2) | | 2 (2) | 1 (1) | 12 |
| **Nausea** | 4 (4) | | |  | 6 (3) | 1 (1) | | | 2 (2) | 1 (1) | 1 (1) |  | | 2 (2) |  | 11 |
| **Vomiting** | 4 (4) | | |  | 3 (3) |  | | | 1 (1) |  | 4(4)† |  | | 2 (2) |  | 10 |
| **Dizziness** | 4 (4) | | |  | 1 (1) |  | | | 3 (3) |  | 2 (2) |  | | 2 (2) |  | 8 |
| **Pruritus** |  | | |  |  |  | | | 1 (1) |  | 2 (2) |  | | 3 (3) |  | 5 |
| **Flushing** | 3 (3) | | |  |  |  | | | 1 (1) | 1 (1) | 2 (2)† |  | |  |  | 3 |
| **Diarrhea** |  | | |  |  |  | | |  |  | 2 (1) | 1 (1) | |  |  | 3 |
| **Somnolence** | 1(1) | | |  | 1 (1) |  | | |  |  | 1 (1) |  | |  |  | 2 |
| **Pruritus generalized** | 2 (2) | | |  | 1 (1) |  | | | 1 (1) |  |  |  | |  |  | 2 |
| **Anxiety** |  | | |  |  |  | | |  | 1 (1) |  |  | |  |  | 2 |
| **Abdominal pain** |  | | |  |  |  | | |  |  | 1 (1) |  | | 1 (1) |  | 2 |
| **Contusion** |  | | |  |  |  | | |  |  |  |  | | 2 (2) |  | 2 |
| **Paraesthesia** |  | | |  |  |  | | | 2 (1) |  |  |  | |  |  | 1 |
| **Anorexia** | 1 (1) | |  | |  |  | |  | |  |  |  | | 1 (1) |  | 1 |
| **Abnormal dreams** |  | |  | |  |  | |  | |  | 1 (1) |  | |  |  | 1 |
| **Arrhythymia** | 1 (1) | |  | |  |  | |  | |  |  |  | |  |  |  |
| **Chest pain** | 1 (1) | |  | |  |  | |  | |  |  |  | |  |  |  |
| **Dermatitis** |  | |  | |  |  | |  | |  | 1 (1) |  | |  |  | 1 |
| **Dermatitis contact** | |  |  | |  |  | 1 (1) | | |  |  | |  |  |  | 1 |
| **Dyspepsia** | |  |  | |  |  |  | | |  | 1 (1) | |  |  |  | 1 |
| **Fatigue** | |  |  | |  |  |  | | |  | 1 (1) | |  |  |  | 1 |
| **Foot fracture** | |  |  | |  |  |  | | |  | 1 (1) | |  |  |  | 1 |
| **Hepatic enzyme increased** | |  |  | |  |  |  | | |  | 1 (1) | |  |  |  | 1 |
| **Hypoxia** | | 1 (1) |  | |  |  |  | | |  |  | |  |  |  |  |
| **Myalgia** | |  |  | |  |  | 1 (1) | | |  |  | |  |  |  | 1 |
| **Pallor** | | 1 (1) |  | |  |  |  | | |  |  | |  |  |  |  |
| **Palpitations** | |  |  | |  |  |  | | |  | 1 (1) | |  |  |  | 1 |
| **Tremor** | |  |  | |  |  | 1 (1) | | |  |  | |  |  |  | 1 |

^a^ Total adverse events for all subjects treated with LY2456302 + fentanyl in Study B (*see Lowe et al. 2014 for total AEs overall)***Supplementary Table S4**. **Pupil diameter measurements recorded from rats and humans receiving naloxone, naltrexone or LY2456302 before mu agonist challenge as shown in Figure 2.**

| **Rodent Pupil Diameter (mm)** | | | | | | | | | | | | | | | | | |
| --- | --- | --- | --- | --- | --- | --- | --- | --- | --- | --- | --- | --- | --- | --- | --- | --- | --- |
|  | **Naloxone** | | | |  | **LY2456302** | | | | | | | | | | | |
|  | **Vehicle** | | **3 mg/kg** | |  | **Vehicle** | | **3 mg/kg** | | **10 mg/kg** | | **30 mg/kg** | | **100 mg/kg** | | **300 mg/kg** | |
| **Time (min)** | **Mean** | **SEM** | **Mean** | **SEM** |  | **Mean** | **SEM** | **Mean** | **SEM** | **Mean** | **SEM** | **Mean** | **SEM** | **Mean** | **SEM** | **Mean** | **SEM** |
| **BL** | 1.2 | 0.08 | 1.33 | 0.1 |  | 1.13 | 0.06 | 1.15 | 0.08 | 1.05 | 0.03 | 1.08 | 0.05 | 1.13 | 0.07 | 1.18 | 0.07 |
| **10** | 1.73 | 0.08 | 1.43 | 0.1 |  | 1.65 | 0.13 | 1.55 | 0.11 | 1.45 | 0.09 | 1.5 | 0.12 | 1.28 | 0.09 | 1.5 | 0.08 |
| **20** | 1.73 | 0.11 | 1.38 | 0.11 |  | 1.48 | 0.1 | 1.55 | 0.07 | 1.55 | 0.08 | 1.43 | 0.08 | 1.48 | 0.14 | 1.53 | 0.09 |
| **30** | 1.7 | 0.12 | 1.45 | 0.12 |  | 1.6 | 0.08 | 1.5 | 0.12 | 1.53 | 0.08 | 1.55 | 0.07 | 1.48 | 0.11 | 1.35 | 0.07 |
| **50** | 1.68 | 0.13 | 1.45 | 0.09 |  | 1.55 | 0.1 | 1.55 | 0.09 | 1.5 | 0.09 | 1.48 | 0.11 | 1.33 | 0.07 | 1.23 | 0.07 |
| **70** | 1.5 | 0.14 | 1.25 | 0.08 |  | 1.35 | 0.12 | 1.33 | 0.1 | 1.3 | 0.07 | 1.35 | 0.11 | 1.3 | 0.08 | 1.45 | 0.1 |
| **90** | 1.43 | 0.08 | 1.28 | 0.11 |  | 1.38 | 0.13 | 1.3 | 0.12 | 1.3 | 0.09 | 1.28 | 0.12 | 1.25 | 0.08 | 1.18 | 0.07 |
|  |  |  |  |  |  |  |  |  |  |  |  |  |  |  |  |  |  |
| **Human Pupil Diameter (mm)** | | | | | | | | | | | | | | | | | |
|  | **Naltrexone** | | | |  |  |  | **LY2456302** | | | | | | | | | |
|  | **Vehicle** | | **50 mg** | |  |  |  | **Vehicle** | | **4 mg** | | **10 mg** | | **25 mg** | | **60 mg** | |
| **Time (min)** | **Mean** | **SEM** | **Mean** | **SEM** |  |  |  | **Mean** | **SEM** | **Mean** | **SEM** | **Mean** | **SEM** | **Mean** | **SEM** | **Mean** | **SEM** |
| **BL** | 6.14 | 0.21 | 6.02 | 0.31 |  |  |  | 6.23 | 0.29 | 6.43 | 0.3 | 6.24 | 0.26 | 5.42 | 0.53 | 5.92 | 0.33 |
| **20** | 3.12 | 0.24 | 5.88 | 0.31 |  |  |  | 3.23 | 0.17 | 3.37 | 0.24 | 3.81 | 0.32 | 3.59 | 0.28 | 4.41 | 0.36 |
| **40** | 3.97 | 0.35 | 5.9 | 0.3 |  |  |  | 4.16 | 0.28 | 4.57 | 0.36 | 4.49 | 0.31 | 4.17 | 0.38 | 4.86 | 0.31 |
| **60** | 4.61 | 0.32 | 6.02 | 0.32 |  |  |  | 4.63 | 0.28 | 5.02 | 0.38 | 5.06 | 0.31 | 4.6 | 0.44 | 5.34 | 0.3 |
| **80** | 4.86 | 0.4 | 5.98 | 0.29 |  |  |  | 4.65 | 0.27 | 5.12 | 0.34 | 5.2 | 0.27 | 4.55 | 0.43 | 5.35 | 0.32 |
| **100** | 4.97 | 0.38 | 6.08 | 0.33 |  |  |  | 4.99 | 0.26 | 5.44 | 0.36 | 5.28 | 0.31 | 4.53 | 0.44 | 5.47 | 0.34 |
| **120** | 5.37 | 0.37 | 6.1 | 0.29 |  |  |  | 5.16 | 0.32 | 5.63 | 0.28 | 5.64 | 0.36 | 4.9 | 0.5 | 5.67 | 0.31 |
| **140** |  |  |  |  |  |  |  | 5.18 | 0.33 | 5.64 | 0.32 | 5.51 | 0.35 | 4.84 | 0.45 | 5.55 | 0.3 |
| **160** |  |  |  |  |  |  |  | 5.32 | 0.39 | 5.67 | 0.38 | 5.58 | 0.35 | 4.99 | 0.47 | 5.68 | 0.33 |
| **180** |  |  |  |  |  |  |  | 5.39 | 0.34 | 6 | 0.25 | 5.75 | 0.33 | 5.04 | 0.52 | 5.71 | 0.28 |
| Abbreviations: BL, baseline; min, minutes; SEM, standard error of the mean. | | | | | | | | |  |  |  |  |  |  |  |  |  |
